# Development and Validation of an HPLC–MS/MS Method for Pioglitazone from Nanocarriers Quantitation in Ex Vivo and In Vivo Ocular Tissues †

**DOI:** 10.3390/pharmaceutics13050650

**Published:** 2021-05-03

**Authors:** Esther Miralles-Cardiel, Marcelle Silva-Abreu, Ana Cristina Calpena, Isidre Casals

**Affiliations:** 1 CCiTUB (Scientific and Technological Centers Universitat de Barcelona), University of Barcelona, 08028 Barcelona, Spain; isidre.casals@ub.edu; 2 Department of Analytical Chemistry, Faculty of Chemistry, University of Barcelona, 08028 Barcelona, Spain; 3 Department of Pharmacy, Pharmaceutical Technology and Physical Chemistry, Faculty of Pharmacy and Food Sciences, University of Barcelona, 08028 Barcelona, Spain; anacalpena@ub.edu; 4 Institute of Nanoscience and nanotechnology (IN2UB), Faculty of Pharmacy and Food Sciences, University of Barcelona, 08028 Barcelona, Spain

**Keywords:** pioglitazone (PGZ), polylactic-*co*-glycolic acid-polyethylene glycol (PLGA-PEG), nanoparticles (NPs), ocular tissues, validation, HPLC-MS/MS

## Abstract

Pioglitazone (PGZ) is an oral anti-hyperglycemic agent, belongs to the class of thiazolidinediones, and is used for the treatment of diabetes mellitus type 2. In recent years, its anti-inflammatory activity has also been demonstrated in the literature for different diseases, including ocular inflammatory processes. Additionally, this drug belongs to Class II of the Biopharmaceutical Classification System, i.e., slightly soluble and highly permeable. The main objective of this study was to validate a new analytical HPLC-MS/MS method to quantify free-PGZ and PGZ from polymeric NPs to conduct nanoparticle application studies loaded with this active ingredient to transport it within ocular tissues. An accurate, sensitive, selective, reproducible and high throughput HPLC-MS/MS method was validated to quantify PGZ in cornea, sclera, lens, aqueous humor, and vitreous humor. The chromatographic separation was achieved in 10 min on a Kinetex C18 column. Linear response of PGZ was observed over the range of 5–100 ng/mL. The recovery of free-PGZ or PGZ from NPs was in the range of 85–110% in all tissues and levels tested. The intra-day and inter-day precision were <5% and <10%, respectively. The extracts were shown to be stable in various experimental conditions in all matrices studied. The range of concentrations covered by this validation is 80–1600 µg/kg of PGZ in ocular tissues. It is concluded that this method can be applied to quantify PGZ for in vivo and ex vivo biodistribution studies related to the ocular administration of free-PGZ and PGZ from nanoparticles.

## 1. Introduction

Pioglitazone (PGZ) is a hypoglycemic therapeutic drug used in the treatment of type 2 diabetes which increases cell sensitivity to insulin via selective stimulation of the nuclear receptor peroxisome proliferator-activator receptor gamma (PPARγ) [1,2]. Studies have shown that the agonist PPARγ receptor is also involved in inflammatory processes [3,4], and PGZ has been found to be effective as an anti-inflammatory drug in animal models of paw edema [5] or acute myocardial infarction [6]. Moreover, PGZ reduced the inflammation in atherosclerosis [7,8], in skin inflammation processes [9,10], and ocular diseases such as uveitis [11] or corneal inflammation [12,13].

Other reported properties of PGZ found in the literature range from lung anti-inflammatory and anti-fibrotic [14], corneal anti-fibrotic [15], corneal neovascularization [16] to dry eye disease treatment [17]. In recent years, research has also been conducted with PGZ for the treatment of Alzheimer’s disease [18,19,20].

As a result of the chemical structure, PGZ is slightly soluble in water and highly permeable in membranes [21]. Due to these characteristics, it is very important to find new vehicles that overcome the solubility problems that restrict its bioavailability when the way of administration for the treatment is not in the form of oral tablets [22,23,24,25].

Nowadays, drug delivery systems that target different organs and tissues are a new challenge. Polylactic-co-glycolic acid (PLGA) is one of the most studied synthetic polymers due to its biocompatibility and biodegradability [26], and it has also been approved by the US Food and Drug Administration (FDA) and the European Medicine Agency (EMA) for drug delivery systems in humans [27]. This polymer allows the development of very small particles with narrow polydispersity. Consequently, it has been used to design nanoparticles (NPs) for biomedical applications as carriers of active ingredients in vaccination, cancer therapies, skin diseases, ocular and neurological disorders [28,29,30,31,32,33,34,35]. However, PLGA is a relatively hydrophobic polymer, and once it is in the blood, it is recognized and phagocyted by macrophages. To avoid this, a modification of its surface using polyethylene glycol (PEG) could be an alternative. PEG is a biocompatible, hydrophilic, nonionic, and approved polymer by the FDA for its use in humans. PEGylation is one of the most used techniques to modify the surface properties of the nanoparticles. In this case, PEGylated NPs take higher polarity and hydrophilicity avoiding aggregation, opsonization and phagocytosis, prolonging systemic circulation time, and facilitating its tissue penetration via interstitial [36].

In ocular therapies, one of the challenges is the effective penetration of the drugs through the eye’s tissue barriers (e.g., corneal, sclera and conjunctiva) to reach targets and to sustain them. Normally, when ophthalmic formulations are used, less than 5% of the drug administered is retained on the ocular surface because of the corneal epithelium barrier and nasolacrimal duct drainage, which means that it is necessary to instill frequently. One of the most successful approaches to overcome this inconvenience is the use of colloidal suspensions of NPs as delivery systems [37,38]. These systems have been described in the literature, showing the effectiveness for ocular delivery principally in form of liposomes [39,40] and polymeric NPs [41,42,43].

In recent years, some studies of our research group have been focused on NPs of PLGA for the treatment of inflammatory disorders, with different therapeutic targets, including the main nanosystems for ocular diseases [33,44,45,46]. The new formulations improved the biopharmaceutical profile of the drugs. In particular, PGZ-NPs of PLGA-PEG were formulated for eye treatment and also for delivery to the brain [12,47]. These nanosystems were optimized, characterized, and their anti-inflammatory activity, as well as their tolerance, were demonstrated for in vitro, ex vivo, and in vivo animal models.

The presented study aimed to develop and to validate an HPLC-MS/MS method following the guidelines of the European Medicines Agency (2019) and the U.S. Food and Drug Administration (2018) for bioanalytical methods validation [48,49] to focus the analysis on the application of free PGZ (solution) and PGZ from NPs in different ocular tissues for future research in the treatment of ocular diseases.

## 2. Materials and Methods

### 2.1. Chemicals and Reagents

PGZ was purchased from Capot Chemical (Hangzhou, China) and diblock copolymer PLGA-PEG 5% (50:50) Resomer^®^ was obtained from Evonik Corporation (Birmingham, AL, USA). Poly(vinyl alcohol) (PVA) Mw 30,000–70,000 87–90% hydrolyzed, dimethylsulfoxide (DMSO) and formic acid were acquired from Sigma-Aldrich-Merck (Darmstadt, Germany). Acetone, acetonitrile and methanol high-performance liquid chromatography (HPLC) gradient-grade were purchased from ITW Reagents (Barcelona, Spain). All reagents used were analytical reagents. Ultrapure water (HPLC grade, >18 MΩ·cm at 25 °C) was obtained using a Milli-Q apparatus from Millipore (Milford, CT, USA).

### 2.2. Chromatographic Conditions

The analysis was carried out using an Agilent 1260 liquid chromatograph (Agilent Technologies, Santa Clara, CA, USA). A reversed-phase Kinetex C18 column (2.6 μm particle size, 50 × 2.1 mm) from Phenomenex (Torrance, CA, USA) was used to perform the chromatographic separation. The gradient between formic acid 0.1% in water (A) and formic acid 0.1% in acetonitrile (B) was the following (t(min), %B): (0, 10), (4, 74), (4.5, 90), (6, 90), (6.1, 10), (10, 10). The column temperature was maintained at 35 °C. The optimization of mobile phase and column selection was based on suitability for the mass detector, effect on the sensitivity of the method and total time required for the analysis. The flow rate was fixed at 0.6 mL/min and the injection volume at 1 µL.

The detection of PGZ was achieved using a triple quadrupole mass spectrometer 4000 QTRAP (Sciex, Framingham, MA, USA) equipped with a TurboV ion source operating in positive ion mode with the following settings: capillary voltage +5500 V, nebulizer gas (N_2_) 50 (arbitrary units), curtain gas (N_2_) 25 (arbitrary units), collision gas (N_2_) medium (arbitrary scale), drying gas (N_2_) 30 (arbitrary units) heated to 550 °C, declustering potential (DP) 100 V, entrance potential (EP) 10 V, collision energy (CE) 35 V and collision cell exit potential (CXP) 12 V.

The use of a triple quadrupole mass spectrometer allowed us to work in multiple reaction monitoring (MRM) mode. The *m/z* transition pairs used for quantitation of PGZ (precursor ion/product ion) were 357.2/134.1 (most sensitive), and 357.2/119.1 for confirmation. The quadrupoles were set at unit resolution. The analytical data were processed with the Analyst software version 1.6.2 (Sciex, Framingham, MA, USA).

### 2.3. Pioglitazone Standards

A stock solution of 1 mg/mL of PGZ was prepared in methanol for subsequent intermediate, spiking, and working standard solutions. The calibration curve was prepared at concentrations of 5, 10, 25, 50, 75 and 100 ng/mL in methanol. Glass vials were used to store the solutions throughout the work.

### 2.4. Biological Material

Ocular specimens were obtained under veterinary supervision from pigs used in surgical University practices at the Faculty of Medicine, according to the Ethics Committee of Animals Experimentation at the University of Barcelona. Pigs (male, weight 30–40 kg) were anesthetized with intramuscular administration of ketamine hydrochloride (3 mg/kg), xylazine (2.5 mg/kg) and midazolam (0.17 mg/ kg). Once sedated, the Propofol (3 mg/kg) was administered by the auricular vein, and immediately afterward they were intubated and maintained under anesthesia inhaled with isoflurane. After chirurgical experimentation, the animals were euthanized (250 mg/kg of sodium pentobarbital was administered through the auricular ear vein under deep anesthesia) and the eyes were immediately extracted and transported to the laboratory in dry ice. Cornea, sclera, lens, aqueous humor and vitreous humor were excised in the laboratory and kept frozen at −20 °C in Eppendorf tubes. The separation between the retina and choroid was dismissed because both expertise and a microscope were needed.

### 2.5. Preparation of Pioglitazone Nanoparticles

PGZ-NPs were obtained by the solvent displacement method described by Fessi et al. [50]. This technique consists of dissolving the polymer and the compound in an organic solvent, being a successful method to deliver the lipophilic drug. This technique is based on two phases: the organic and the aqueous phase. Ten mg of PGZ were solubilized in 0.5 mL of DMSO and 75 mg of PLGA-PEG were dissolved with 5 mL of acetone. Both organic solutions were mixed followed by sonication in an ultrasonic bath (100 w) for two minutes. This organic phase was added drop by drop, gently mixing, into 10 mL of an aqueous solution of PVA 1.5%. The NPs dispersion was concentrated to 10 mL under reduced pressure (Vacuubrand PC 2001 Vario, Wertheim, Germany). The PGZ-NPs suspensions were stable in the fridge for 6 weeks. Parameters such as size, polydispersity index (PDI) and Zeta Potential (ZP) were evaluated to confirm the stability with a Zetasizer Nano ZS (Malvern Instruments, Malvern, UK). Additionally, the encapsulation efficiency (EE) was determined by the indirect method [47].

### 2.6. Pioglitazone Solutions for Biological Matrix Spiking

The suspension of PGZ-NPs containing about 1 mg/mL of PGZ (2.5) was diluted in water for spiking into ocular tissues (cornea, sclera, aqueous humor, vitreous humor, lens). The exact amount of PGZ in the suspension NPs was quantified in order to calculate the accuracy and precision of the method when PGZ-NPs were spiked into the tissues. The chromatographic conditions for PGZ quantitation in the NPs were adapted from previous studies: liquid chromatography with reverse phase column C18, eluent acetonitrile-ammonium acetate buffer with ultraviolet detection at 268 nm [9]. Free-PGZ solution (1 mg/mL) was diluted in methanol to prepare spiked samples (2.3).

### 2.7. Extraction Procedure

Lens, aqueous and vitreous humor were vortex-mixed previously. Sclera and cornea were minced and thoroughly perforated using a needle to help the penetration of the extraction solution. For cornea, sclera, and lens, the taken weight was about 125 mg; for aqueous and vitreous humor, the weight was about 250 mg. The samples were weighed into amber glass vials and were extracted with 2 mL of methanol using an ultrasonic bath for 30 min. The solution was centrifuged at 10,000 rpm for 10 min at room temperature. The resultant supernatants were filtered through nylon filters of 0.22 µm and injected into the HPLC-MS/MS system. The stability of the extracts showed that the supernatants could be collected and stored at −20 °C until use (three months tested).

### 2.8. Method Validation

The method was validated according to the bioanalytical method validation guidelines of the EMA and the FDA and considering the acceptance criteria recommended for the following validation parameters: selectivity–specificity, matrix effect, calibration curve (response function), limits of quantification and detection, accuracy, precision, recovery, carry-over, dilution integrity and stability [48,49].

#### 2.8.1. Selectivity and Specificity: Matrix Effect

Blank tissues were analyzed to determine the selectivity and specificity of the method. Any interference from unwanted tissue components at the elution time of PGZ was evaluated.

The matrix effect is defined as an alteration of the analyte response due to interfering components in the sample. The effect of tissue components on the ionization of PGZ was determined by comparison of the response of three replicates of a free-PGZ working solution prepared in methanol and free-PGZ working solutions prepared in the blank extract for each tissue. The PGZ concentration was calculated with the calibration curve. Along with the validation procedure, the matrix effect was also evaluated for accuracy and precision data.

#### 2.8.2. Recovery

Weighed tissues were spiked with free-PGZ and PGZ-NPs at three levels of concentration to achieve the final concentrations in the extract of about 10 (low), 20 (medium) and 100 (high) ng/mL, *n* = 3 each level. As free-PGZ was spiked from small volumes (10–20 µL) of standard solutions prepared in methanol, to reduce pipetting errors the aliquot added was weighed with a minimum precision of 0.1 mg. Samples spiked with the free-PGZ standard solution were extracted after 60 min waiting time at room temperature and those spiked with PGZ-NPs were extracted after 3 h waiting time at room temperature to let them interact with the tissue and better simulate incurred residues. The extracts were kept at −20 °C until HPLC-MS/MS analysis.

#### 2.8.3. Dilution Integrity

The sample dilution effect was also evaluated to ensure that PGZ concentrations beyond the upper limit of concentration of the calibration range could be correctly determined after dilution of the extract with methanol. Three replicates of spiked tissues were used to investigate the intra-day precision and accuracy of a 50-fold sample dilution. The ocular tissues were spiked to achieve the final concentration of PGZ in the injected extract of about 50 ng/mL. Both addition of free-PGZ and PGZ-NPs were assayed. This concentration corresponded to about 2000 µg PGZ/kg for aqueous and vitreous humor, and 4000 µg PGZ/kg for lens, sclera and cornea.

#### 2.8.4. Calibration Curve

The calibration curve was prepared using six calibration standards defined in Section 2.3. The chromatographic area of PGZ against the concentration of the calibration standards was plotted to construct the calibration curve. The linearity of the method was evaluated by weighted linear regression analysis, with 1/x^2^ as the weighting factor, using the least-squares method [51]. The acceptance criteria for the back-calculated standard concentration were ±15% deviation from the theoretical value. The calibration curve (six standards) was prepared using fresh standards in each assessment.

#### 2.8.5. Accuracy and Precision

The spiked levels of PGZ concentration corresponded to approximately 160, 320 and 1600 µg/kg for lens, cornea and sclera. For aqueous humor and vitreous humor, the spiked levels of PGZ concentration corresponded to approximately 80, 160 and 800 µg/kg. The closeness of mean results determined by the method to the spiked concentration of the analyte and the repeatability was evaluated in terms of recovery. Three sets of three different levels of tissues spiked with free-PGZ were prepared and quantified on three separate days to determine the inter-day and intra-day precision and the accuracy of the method. Samples spiked with PGZ-NPs were prepared and quantified for accuracy and intra-day precision (*n* = 3) at the three levels of concentration.

The acceptance criteria for accuracy of the data should be within ±15% (recovery 85–115%). For the limit of quantitation (LOQ) the accuracy acceptable limit of deviation is ±20% (recovery 80–120%). The precision around the theoretical value should not cross 15% of the coefficient of variation (CV) and 20% at LOQ [48,49].

#### 2.8.6. Limits of Quantification (LOQ) and Detection (LOD)

The LOQ is defined as the lowest concentration that can be measured with an intra-day and inter-day precision (expressed as the percentage of the coefficient of variation, CV) that must not exceed 20%, and accuracy (expressed as the percentage of the deviation from nominal concentration, bias) that must be within ±20%. The LOQ was evaluated in three replicates on a single day and three different days for all studied matrices.

The limit of detection (LOD) consists of the lowest concentration whose respective signal can be dependably distinguished from the background level. The LOD was assessed by analyzing successive dilutions of blank extract of tissues with added known amounts of PGZ. See Section 3.2.9 for more details.

#### 2.8.7. Stability Experiments

The obtained extracts from the recovery and precision study were kept at −20 °C till the day of the HPLC-MS/MS analysis because of the availability of the equipment. The long-term stability evaluation was deduced from the obtained results of accuracy and precision studies (three different days). One of the sets was injected three months after the extraction, the other two sets after 9 and 15 days, respectively.

Extracts of each tissue spiked at approximately the medium level of concentration were injected to determine the stability of pioglitazone in various predetermined conditions. The post-preparative stability of the samples was determined after comparison of the response of immediate injection of the extracted samples to that of the re-injected samples after keeping them in the refrigerated autosampler for 24 h. The post-preparative stability of the drug was assessed, taking into account the time that the samples could remain in queue in the chromatograph and, eventually, any failure of the chromatograph in a 24 h period. Short-term stability was determined after placing the extracts for eight hours on the bench top at room temperature. The period for the short-term stability study was determined based on the possible time spent for a batch sample analysis. The relative stability was calculated by considering the initial area of PGZ as 100%.

### 2.9. Bioavailability Experiment: In Vivo

To investigate the ocular bioavailability and disposition of PGZ, pig eyes were treated with topical administration of 0.05 mL of PGZ-NPs suspension of a concentration of about 1 mg/mL of PGZ. After four hours the pig was euthanized. Cornea, lens, sclera, aqueous and vitreous humors were processed according to the validated method.

## 3. Results

### 3.1. Nanoparticle Preparation

The PGZ-NPs were synthesized by displacement technique, showing a particle size around 200 ± 2.5 nm, PDI of 0.2 ± 0.03 and a ZP of −15.0 ± 1.3 mV. Moreover, the percentage of EE was around 92.1 ± 3.2%. These NPs were shown to be stable in the fridge for six weeks.

### 3.2. Method Validation

#### 3.2.1. Selectivity and Specificity

**Figure 1:**
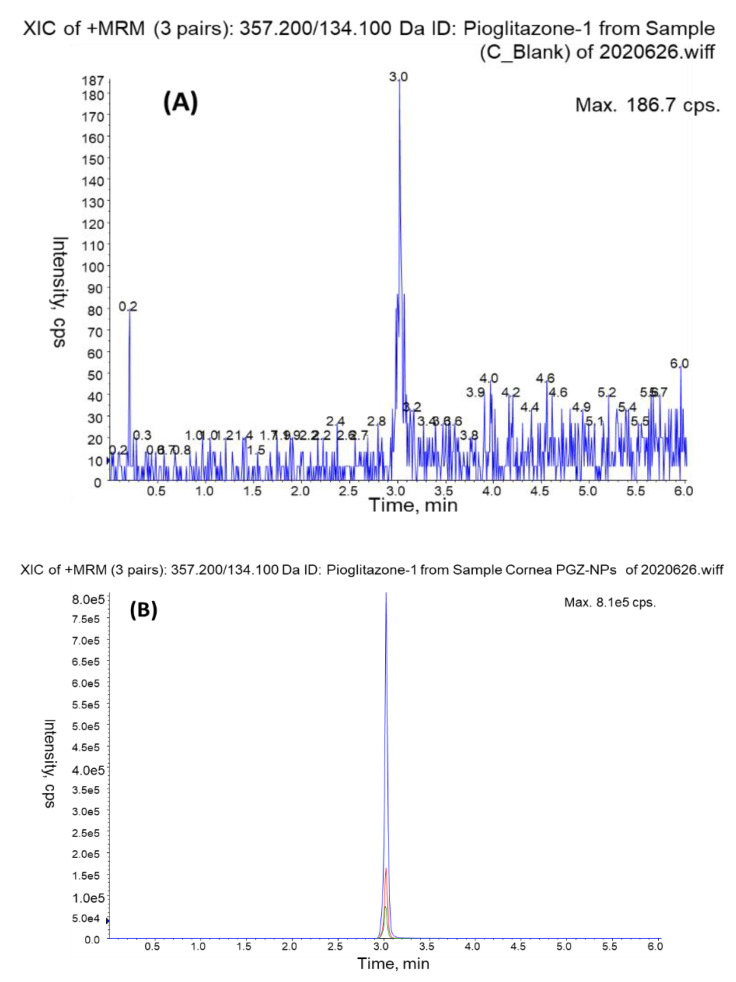
MRM chromatograms of cornea blank extract (**A**) and a cornea spiked with PGZ-NPs at three levels of concentration: blue (high level), red (medium level), green (LOQ) (**B**).

**Figure 2:**
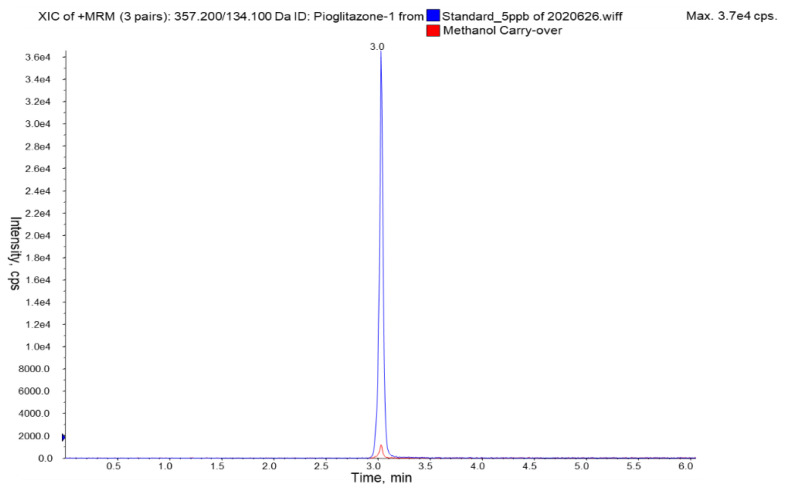
MRM chromatograms of methanol carry over (red) and PGZ standard of 5 ng/mL (blue).

The analysis of blank samples (matrix samples processed without the addition of PGZ) showed no interfering signals in chromatograms of the blank extracts. The retention time for PGZ was about 3 min and there were no interfering matrix components when determining PGZ in any of the tissues of the assay. Typical MRM chromatograms of PGZ found in cornea and PGZ standard (5 ng/mL) are shown in Figure 1 and Figure 2, respectively.

#### 3.2.2. Matrix Effect

Despite HPLC-MS/MS standing out for its high selectivity and specificity, the composition of the matrix is very important and must be taken into account. The matrix composition could be responsible for increasing or decreasing the signal of the ions being studied.

It was assessed that the matrix components of the tissues did not change the PGZ response concerning the analyte in a matrix of methanol as the variation found was less than ±5% (Table 1). Values shown in Table 1 are the average of three replicates. For all matrices, the results are in accordance with the acceptance criteria: CV not exceeding 15% for precision and bias within ±15% for accuracy. The external calibration method could, therefore, be used.

#### 3.2.3. Recovery

The concentration of the spiked sample extracts was calculated from the PGZ calibration curve (Table 2, Table 3 and Table 4). Average extraction recoveries for PGZ, *n* = 3, were in the range 85–110% (bias −15% to +10%). For all matrices, the results are in accordance with the acceptance criteria: CV not exceeding 15% for precision and bias within ±15% for accuracy. The method applies to Free-PGZ and PGZ from NPs. Recovery was obtained as R (%) = ([PGZ] found/[PGZ] added) × 100.

#### 3.2.4. Calibration Curve

**Figure 3:**
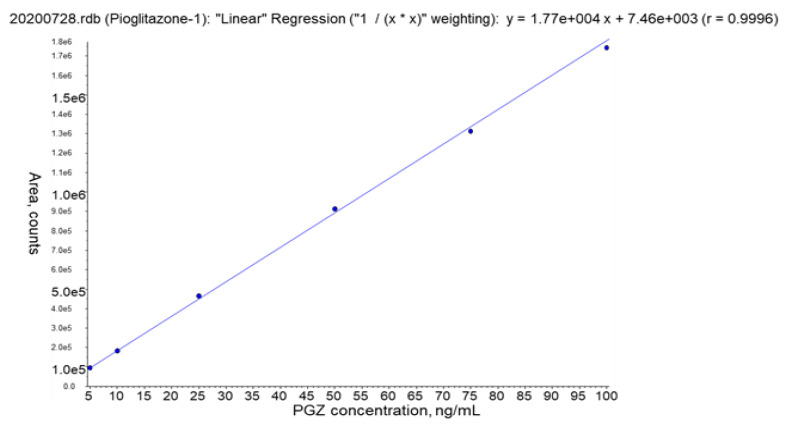
Example of PGZ calibration curve (Linear Regression “1/x^2^” weighting: y = 1.77e + 004 + 7.46e + 003 (r = 0.9996)).

The calibration curve was linear in the PGZ concentration range of 5–100 ng/mL with a correlation coefficient R^2^ > 0.99. The calibration curve was freshly prepared for each chromatographic run. The accuracy found of back-calculated concentration during the different days of analysis was within 91–108% (accepted ±15% in terms of bias) [48,49]. Figure 3 shows a calibration curve obtained during validation experiments.

#### 3.2.5. Accuracy and Precision

Experimental concentrations of spiked samples should not deviate more than ±15% from their nominal concentrations. Intra-day and inter-day accuracies (Table 2, Table 3 and Table 4) given by bias (100-Recovery%) varied between −15% for lens and sclera at some added levels to +10% for the lens at the 50-fold diluted level, but no bias was observed with concentration. The results shown in Table 2, Table 3 and Table 4 indicate that the intra-day and inter-day precisions, given as CV (%), did not exceed 5% and 10%, respectively. The acceptance criteria defined by the EMA and FDA were fulfilled at the assessed concentration levels.

#### 3.2.6. Dilution Integrity

The dilution integrity of the samples to demonstrate that a sample dilution procedure, when required, will not impact the accuracy and precision of the measured concentration of the analyte, was investigated in all matrices. It was demonstrated that a 50-fold dilution of a spiked sample can be applied when the concentration of the sample is higher than the upper limit of the calibration curve (Table 1 and Table 3).

**Figure 4:**
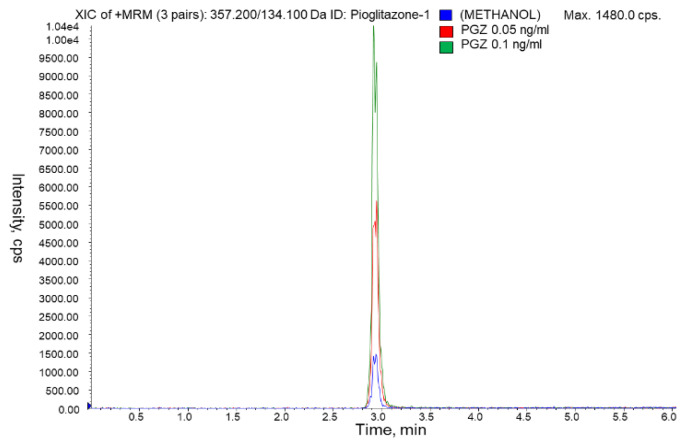
MRM chromatograms of blank-methanol (blue) and PGZ standards of 1 ng/mL (green) and 0.05 ng/mL (red).

In contrast, if the concentration in the extract of real samples was below the lower limit of the calibration curve, a new calibration curve could be constructed with lower concentration standards. Minor modifications like increasing the injection volume could be assayed to get more intensity because it was found that standards of PGZ of 0.05 ng/mL provided a signal distinguishable from the blank (Figure 4). However, according to international guidelines, if method conditions were modified, the results should be confirmed with a partial validation, comprising at least accuracy and precision determination.

#### 3.2.7. Carry-Over

Carry-over was assessed during method development and validation and it was observed that it was unavoidable. At the beginning of the trial, we had no signal attributable to PGZ in the blank ionograms. When we started injecting solutions containing PGZ, small ion signals of ions attributable to this molecule began to appear in the blanks, and was impossible to remove them even by cleaning the ionization source of the spectrometer. Methanol was injected at the beginning of the sequence, after the standards calibration curve and following the highest levels of spiked samples. Nevertheless, the intensity of the carry-over signal was practically constant during the batch sequence and with low variation from one day to another. No concentration effect, therefore, was observed on the carry-over signal. The carry-over signal in the blank of methanol was found to be between 1% and 4% of the analyte response at the limit of quantitation level (10 ng/mL). The extracts of blank tissues showed the same peak intensity as the blank of methanol. These signal values are in the low range of those accepted in the harmonized guidelines of validation methods because they recommend that carry over should not exceed 20% of the LOQ [48,49]. Figure 2 shows a chromatogram of the carry-over signal and a PGZ standard of 5 ng/mL. Accuracy and precision data confirmed that the presence of the carry-over effect did not affect the results.

#### 3.2.8. Stability

**Figure 5:**
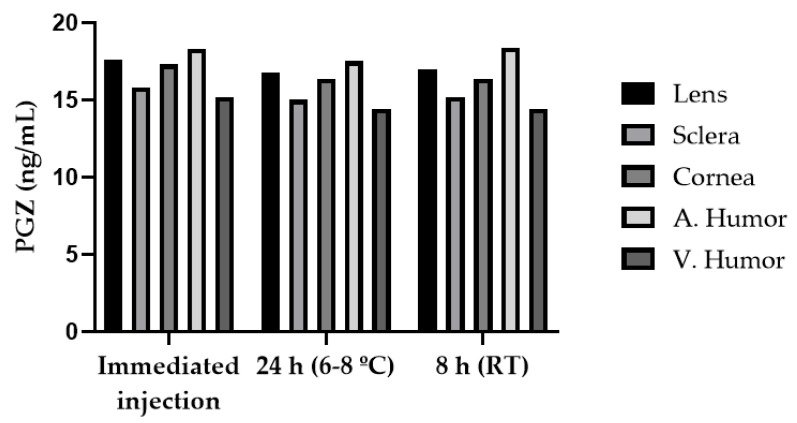
Stability data of PGZ in extracts of the tissues.

Long-term (three months) sample stability at −20 °C was confirmed based on the obtained results for accuracy (bias ±15%) and precision (CV < 15%), which were found to be within the acceptable limits. The data for refrigerated stability of the extracts obtained from in-injector stability (24 h), and short-term stability (8 h) of the extracts at room temperature indicated that the variation of the response of the extracts was less than ±5% (Figure 5). The accuracy of back-calculated concentrations in the calibration curve was accepted to be ±15%, therefore, the variation found in the injection of stability of extracts should be accepted.

#### 3.2.9. Limits of Quantification (LOQ) and Detection (LOD)

The LOQ was experimentally accomplished for all tissues and the intra- and inter-day precision and accuracy results are reported in Table 2, Table 3 and Table 4. The LOQ was established as 160 µg/kg for sclera, cornea and lens, and 80 µg/kg for aqueous humor and vitreous humor (10 ng/mL in extract).

The LOD is defined as the concentration that provides a good peak visualization with the lowest signal-to-noise ratio (S/N) possible [52]. PGZ solutions of 0.05 ng/mL in tissue extracts were injected and the signal was more than twice in height the carry-over signal for all the examined tissues and around three times in the area. Taking as background noise the carry over the peak and the criterion of estimating signals three times higher in area than the background noise, the LOD was established in the range 0.4–0.8 µg/kg of PGZ in tissues (Figure 4).

### 3.3. In Vivo Bioavailability Study

**Figure 6:**
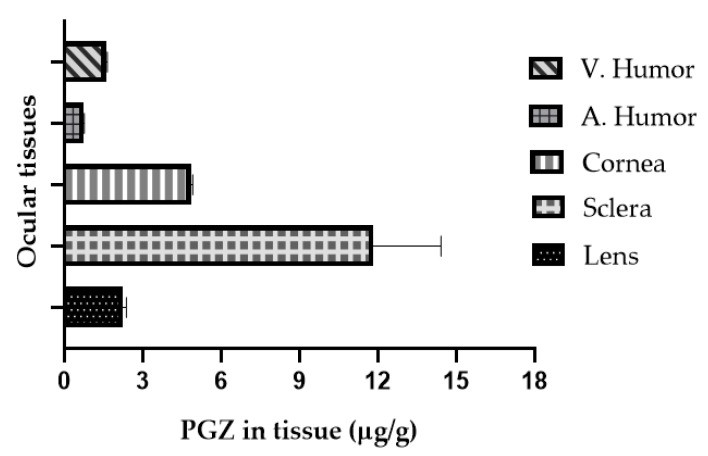
Analysis of samples: PGZ found in tissues after PGZ-NPs ocular administration (*n* = 3).

The method proposed herein was used in a pilot study to quantify PGZ in lens, cornea, sclera, aqueous and vitreous humors, after eye’s administration in form of PGZ-NPs. The method was sensitive enough to quantify PGZ in the tissues, demonstrating its applicability. The results presented in Figure 6 show that the maximum concentration was found in the sclera followed by the cornea. Aqueous humor was the tissue where the concentration was lowest. According to the validated method, extracts of lens, vitreous humor, cornea and sclera had to be diluted to fall into the curve concentration range. A dilution 1/20 was necessary for sclera and cornea extracts, and a dilution of 1/2 for vitreous humor and lens extract.

## 4. Discussion

PGZ-NPs were successful produced using the displacement technique; this method provided small particles with good homogeneity and a negative charge of −15.0 mV. This negative value is indicative of the stability of these systems in solution. Moreover, a higher percentage of EE was found (around 92%) for PGZ into the NPs. According to these physicochemical characteristics, these systems could be suitable for the ocular application. Previous studies have evaluated PLGA-NPs as ocular drug carriers, and it was demonstrated that the ocular penetration and permeability of the drugs were improved when they were encapsulated into the polymer [33,43,44,45,46]. The sensitivity of HPLC-UV methods did not allow the determination of PGZ in real biological samples with a simple extraction nor probably with preconcentration strategies. Therefore, HPLC-MS was an alternative technique to consider. Previously published data have shown that HPLC-MS has been used for PGZ quantitation in liquid biological samples (urine, plasma, serum) [53,54]. However, this is the first validated method using biological ocular tissues. This method was validated for free-PGZ and PGZ from NPs spiked in ocular tissues, and treated tissues were also analyzed.

The method was validated in the range 80–1600 µg/Kg of PGZ extracted from the tissues with good linearity between 5–100 ng/mL of PGZ standards. The extraction recovery was satisfactory for free-PGZ and PGZ from NPs with recoveries found in the range of 85–110%. The reproducibility of the method obtained through the inter-day and intra-day precision was CV < 10% and CV < 5% respectively. Moreover, it was demonstrated that a dilution of the extract, if necessary, made it possible to quantify PGZ with no impact on the accuracy and precision. The LOD and LOQ achieved in cornea, sclera, and lens were on the orders of 0.8 µg/kg and 160 µg/kg respectively. LOD and LOQ in eye humors were in the orders of 0.4 µg/kg and 80 µg/kg respectively. The sensitivity was slightly improved compared to data found in the literature [54]. All the experimental data agree with the guidelines for bioanalytical method validation [48,49].

Significant levels of PGZ were found in eye tissues after 4 h of PGZ-NPs installation (Figure 6), which means that the formulation permeated and remained in the tissues. Aqueous humor, which is in contact with the cornea, was the tissue where the amount was about one order of magnitude lower with respect to the sclera, although PGZ remained. Vitreous humor, situated behind the sclera, also presented a good level, indicating that the formulation permeated through the sclera. These data are in accordance with a previous study in which PGZ accumulated more in sclera than in the cornea [12], probably due to the hydrophilic characteristics of sclera. This new biodistribution experiment supports previous results, and this validated method covers the needed concentration range for studying different parts of the eye after treatment with PGZ. The formulation developed perhaps could be useful for the treatment of ocular inflammation with the ability to sustain PGZ in the tissues and to reach the posterior segment.

Taken together, these results indicate that an accurate, sensitive, selective, reproducible, and high-throughput HPLC-MS/MS method was developed and fully validated for the quantitative determination of PGZ from solution and NPs over a wide concentration range in different eye tissues. This method has the advantage of easy sample preparation, thus reducing assay time. The sensitivity and selectivity achieved for the detection of PGZ with respect of HPLC-UV make it suitable for analyzing very low levels of concentration in complex biological matrices. Moreover, HPLC-MS/MS allows the unambiguous identification of PGZ. The method can be easily applied to in vivo and ex vivo biodistribution studies related to the ocular administration of PGZ-NPs. Moreover, it opens the applicability to other types of biological matrices for preclinical or clinical use.

## Figures and Tables

**Table 1:** Matrix effect in the response of PGZ (*n* = 3).

PGZ Matrix Condition	Mean Found (ng/mL)	SD	CV (%)	Variation (%)
Methanol	51.8	0.56	1.07	-
Sclera extract	54.6	1.65	3.02	+5.34
Cornea extract	53.5	1.47	2.75	+3.12
Lens extract	52.2	0.55	1.06	+0.69
Aqueous humor extract	52.5	0.20	0.38	+1.34
Vitreous humor extract	49.0	1.10	2.24	−5.27

**Table 2:** Absolute recovery (values in mean percentages) of Free-PGZ determined at low, medium and high concentration, and 50-fold diluted sample. Intra-day results (*n* = 3).

Level of Concentration in Extract	Recovery (%)				
ng/mL	Lens	Cornea	Sclera	Aqueous Humor	Vitreous Humor
10	89.0 ± 1.9 * CV (%) = 2.1	96.9 ± 0.5 CV (%) = 0.5	97.7 ± 3.3 CV (%) = 3.4	92.2 ± 0.4 CV (%) = 0.4	89.5 ± 1.6 CV (%) = 1.7
20	85.0 ± 1.4 CV (%) = 1.7	100.9 ± 2.2 CV (%) = 2.2	98.5 ± 2.7 CV (%) = 2.7	91.6 ± 1.2 CV (%) = 1.3	89.4 ± 0.4 CV (%) = 0.4
100	85.2 ± 0.5 CV (%) = 0.6	93.9 ± 3.2 CV (%) = 3.4	88.4 ± 2.7 CV (%) = 3.1	86.4 ± 1.1 CV (%) = 1.3	86.7 ± 1.7 CV (%) = 1.9
2500	109.6 ± 3.8 CV (%) = 3.5	85.0 ± 0.7 CV (%) = 0.8	88.0 ± 0.4 CV (%) = 0.4	97.2 ±1.8 CV (%) = 1.9	99.6 ± 0.4 CV (%) = 0.4

* Mean ± SD.

**Table 3:** Absolute recovery (values in mean percentages) of Free-PGZ determined at low, medium and high concentration. Inter-day accuracy and precision data (*n* = 9).

Level Concentration in Extract	Recovery (%)				
ng/mL	Lens	Cornea	Sclera	Aqueous Humor	Vitreous Humor
10	93.1 ± 4.9 * CV (%) = 5.2	97.8 ± 1.5 CV (%) = 1.5	89.7 ± 7.9 CV (%) = 8.8	92.3 ± 8.4 CV (%) = 9.1	98.3 ± 6.7 CV (%) = 6.8
20	91.8 ± 5.3 CV (%) = 5.8	97.4 ± 3.1 CV (%) = 3.2	89.1 ± 8.2 CV (%) = 9.2	92.5 ± 5.7 CV (%) = 6.2	98.5 ± 6.9 CV (%) = 7.0
100	87.8 ± 2.2 CV (%) = 2.4	93.0 ± 6.9 CV (%) = 7.4	85.0 ± 3.1 CV (%) = 3.6	87.7 ± 4.8 CV (%) = 5.4	92.3 ± 4.3 CV (%) = 4.7

* Mean ± SD.

**Table 4:** Intra-day accuracy and precision data (*n* = 3) for tissues spiked with PGZ-NPs: low, medium, high concentration levels, and 50-fold diluted sample.

Tissue	Nominal Concentration in Extract				
	Added (ng/mL)	Found (ng/mL)	SD	CV (%)	Recovery (%)
Lens	8.4	7.2	0.2	2.7	85.9
	17.5	18.9	0.6	3.0	108.0
	87.7	90.2	1.9	2.2	102.8
	2094	2294.6	79.2	3.5	109.6
Cornea	8.4	7.8	0.3	4.0	93.0
	16.8	15.5	0.2	1.1	92.1
	84.2	76.0	1.4	1.8	90.2
	2094	1779.1	13.6	0.8	85.0
Sclera	8.4	7.8	0.3	3.7	92.0
	16.8	16.0	0.4	2.4	94.9
	84.2	74.6	0.9	1.3	88.5
	2094	1842.8	7.6	0.4	88.0
Aqueous	7.4	6.4	0.3	4.2	87.4
Humor	14.8	12.6	0.2	1.3	85.2
	73.8	62.9	0.4	0.7	85.3
	1931	2139.0	40.1	1.9	110.8
Vitreous	7.4	7.3	0.4	6.1	99.1
humor	14.8	14.6	0.3	2.0	98.8
	73.8	70.7	1.8	2.5	95.8
	1937	1925.3	7.2	0.4	99.4

## Data Availability

Not applicable.
